# Effect of Carbohydrate Supplementation on Investment into Offspring Number, Size, and Condition in a Social Insect

**DOI:** 10.1371/journal.pone.0132440

**Published:** 2015-07-21

**Authors:** Bill D. Wills, Cody D. Chong, Shawn M. Wilder, Micky D. Eubanks, David A. Holway, Andrew V. Suarez

**Affiliations:** 1 Department of Animal Biology, University of Illinois, Urbana, Illinois, United States of America; 2 School of Integrative Biology, University of Illinois, Urbana, Illinois, United States of America; 3 Department of Entomology, Texas A&M University, College Station, Texas, United States of America; 4 Divisison of Biological Sciences, University of California at San Diego, La Jolla, California, United States of America; 5 Department of Entomology; Program in Ecology, Evolution and Conservation Biology, University of Illinois, Urbana, Illinois, United States of America; University of Sheffield, UNITED KINGDOM

## Abstract

Resource availability can determine an organism’s investment strategies for growth and reproduction. When nutrients are limited, there are potential tradeoffs between investing into offspring number versus individual offspring size. In social insects, colony investment in offspring size and number may shift in response to colony needs and the availability of food resources. We experimentally manipulated the diet of a polymorphic ant species (*Solenopsis invicta*) to test how access to the carbohydrate and amino acid components of nectar resources affect colony investment in worker number, body size, size distributions, and individual percent fat mass. We reared field-collected colonies on one of four macronutrient treatment supplements: water, amino acids, carbohydrates, and amino acid and carbohydrates. Having access to carbohydrates nearly doubled colony biomass after 60 days. This increase in biomass resulted from an increase in worker number and mean worker size. Access to carbohydrates also altered worker body size distributions. Finally, we found a negative relationship between worker number and size, suggesting a tradeoff in colony investment strategies. This tradeoff was more pronounced for colonies without access to carbohydrate resources. The monopolization of plant-based resources has been implicated in the ecological success of ants. Our results shed light on a possible mechanism for this success, and also have implications for the success of introduced species. In addition to increases in colony size, our results suggest that having access to plant-based carbohydrates can also result in larger workers that may have better individual fighting ability, and that can withstand greater temperature fluctuations and periods of food deprivation.

## Introduction

The study of life history traits is central to the fields of ecology, behavior, and evolution [1–3]. Life history theory explores investment into key biological characteristics that figure directly into the reproductive success and survival of an organism (e.g., size at birth, age and size at maturity) [1, 4]. One of the major tenets of life history theory is that finite resources are differentially allocated to traits associated with growth, defense, and reproduction [1, 5–7]. Consequently, investment in different life history traits is subject to tradeoffs associated with resource allocation; resources devoted to one function cannot be allocated to another (e.g., offspring size versus offspring number) [1, 4, 5].

Body size represents a key life history trait that is frequently used for both intraspecific and interspecific comparisons [8–12]. For eusocial organisms, a colony’s investment in the size and condition of its individual members can influence colony fitness through colony maintenance, survival, and reproduction [13–15]. For example, body size affects worker metabolism [16, 17], thermal tolerance [18, 19], locomotion [20, 21], longevity [16, 22], and foraging/prey selection [23–25]. Consequently, body size plays an important role in determining how organisms interact with the biotic and abiotic environment [19, 25–27].

Relative to solitary species, the study of life history traits in eusocial organisms is complicated by their reproductive division of labor. In social insects, reproduction is dominated by the queen caste, while the majority of other tasks within a colony are performed by the worker caste, often made up of sterile individuals. This separation of reproductive and non-reproductive individuals within a colony can influence tradeoffs that may constrain the evolution of life history traits in solitary organisms. For example, ant workers rarely display morphological characters related to dispersal, mating, and reproduction. Subsequently, fewer resources are diverted to these structures during larval development. Additionally, ants often display pronounced variation in body size (e.g. worker polymorphism) both within and among colonies [13, 28, 29]

Variation within and between colonies in investment into the number, size and quality of workers may result from plastic responses to colony needs (e.g., defense) and the availability of food resources [15, 30, 31]. Furthermore, as holometabolous insects, differences in initial reproductive investment (e.g., egg size) and larval feeding prior to pupation can have dramatic effects on adult worker body size [32–34]. An increase in the total amount of food resources available to a colony can increase worker size [26, 23, 35, 36], alter body size distributions [33, 37, 38], and increase colony size [39, 40]. Colonies are more likely to produce larger workers as they increase in size and age, further influencing worker body size variation [41, 42].

Body size is not only influenced by the overall amount of food received, but also by diet composition. For example, colonies reared on carbohydrate-rich diets have greater worker and brood production compared to those deprived of carbohydrates [39, 40, 43]. Ants can obtain carbohydrate-rich resources directly from plants (e.g., extrafloral nectaries), or indirectly from honeydew-producing insects (e.g., Hemiptera). Moreover, there has been a growing interest in linking the ecological success of ants to their access to plant-based carbohydrate resources [44, 45]. Access to carbohydrate resources may be particularly important for the success of introduced ant species by influencing colony growth and establishment [46, 47, 48, 49, 40, 50]. The red imported fire ant (*Solenopsis invicta*), for example, often monopolizes carbohydrate-rich resources from honeydew producing insects in introduced populations [40, 49, 51], and does so at greater frequencies than in native populations [52]. This shift in resource assimilation may enhance colony growth and performance [40, 49, 52, 53], and in turn, influence the outcome of direct and indirect competition [35, 54].

In this study, we manipulated access to artificial extrafloral nectar in laboratory colonies of *S*. *invicta* to test how the amino acid and carbohydrate components of nectar influence colony investment in worker number, mean worker body size, and fat content. Wilder and colleagues [40] reported that access to plant-based carbohydrates resulted in increased brood production and a near doubling of dry biomass of workers over a 60-day period. To determine the consequences of a doubling of dry biomass and increase in brood production on *S*. *invicta* workers, we quantified how access to carbohydrates altered investment in worker number, body size, and percent fat mass. While previous work has examined the role of diet on worker production or biomass [39, 40, 43], few studies simultaneously examine multiple metrics of colony investment into worker production (e.g., worker number, size, and quality). We also tested whether the macronutrient components of extrafloral nectar (carbohydrates, amino acids, or both) shift the distribution of worker body sizes within a colony and if there are potential tradeoffs between investment into worker quality or number across diet treatments.

## Materials and Methods

### Study System

We examined the effects of carbohydrates and amino acid supplementation on colony investment in worker number, condition and size in the red imported fire ant (*Solenopsis invicta*), a widespread introduced species native to northern Argentina and southern Brazil [55]. Workers are continuously polymorphic with a wide range of body sizes (head widths 0.45 mm– 1.50 mm) and a distribution skewed towards smaller workers [56]. Although largely omnivorous [52, 57–59], *S*. *invicta* frequently consumes carbohydrate-rich resources (e.g., hemipteran honeydew) [49, 59].

### Colony Collection and Experimental Design

We excavated colonies of polygyne *S*. *invicta* from the campus of Texas A&M University (College Station, Brazos County, Texas, USA) in the spring of 2008 and 2009 [40]. We slowly flooded field-collected material with water to separate workers, brood, and queens from the soil. Each field colony was split into four experimental subcolonies (one replicate for each treatment) consisting of two queens, ~ 50 brood and 1 g wet mass of workers (0.3456 ± 0.009 g dry mass or 1192 ± 62 individuals). Subcolonies were housed in plastic containers (56 cm length x 40 cm width x14 cm height) lined with fluon, and provided with a vial of water and a darkened petri dish lined with plaster for a nest. The plaster substrate was moistened twice a week. We provided all colonies with two freshly killed crickets, *Acheta domesticus*, three times per week, which was *ad libitum* prey for colonies used in these experiments, allowing colonies to balance their protein to carbohydrate intake [43]. Colonies were maintained on a 12:12 light:dark photoperiod with 40–70% humidity and a daily temperature cycle that included 8 hours during daylight at 32°C and 16 hours at 24°C. The experiments were run for 60 days after which time we froze all experimental colonies.

To test the effects of increased access to a nectar resource, and its constituent components, on investment into worker number and condition, we supplemented the colonies with a 5 mL vial of one of four randomly assigned treatments: “water” (*n* = 12); “carbohydrate”—a solution containing only the carbohydrate component of extrafloral nectar (*n* = 13); “amino acid”—a solution containing only the amino acid component of extrafloral nectar (*n* = 14); “nectar”—a solution containing both the carbohydrate and amino acid components of extrafloral nectar (*n* = 15). The carbohydrates and amino acids mimicked the chemical composition of extrafloral nectar of *Passiflora* sp. [60] and consisted of 1 L of water mixed with carbohydrates (108 g sucrose, 90 g glucose, 53 g fructose) and amino acids (0.0232 g aspartic acid, 0.512 g glutamine, 0.0404 g glutamic acid, 0.0194 g histidine, 0.0436 g isoleucine, 0.04 g leucine, 0.118 g phenylalanine, 0.368 g proline, 0.0704 g tryptophan, and 0.1122 g tyrosine). This artificial nectar recipe has carbohydrate (251 g/L) and amino acid (1.3 g/L) concentrations similar to a wide range of extrafloral nectars (carbohydrate, mean = 222, median = 183; amino acid, mean = 3.4, median = 1) [61]. We only included carbohydrates and amino acids in our artificial extrafloral nectar as other components, such as volatiles to attract pollinators, are not likely needed for the nutritional demands of consumers [61]. We replaced the vials with the experimental treatments twice each week.

### Measurements

At the end of the experiment, we counted the total number of workers and took head measurements from 200 individuals per colony to determine mean size and body size distributions. To minimize potential biases in worker selection, we spread the entire worker population of each colony across the surface of a 150 mm diameter petri dish. An assistant who was blind to the design and predictions of the experiment, randomly selected workers from each of five equally sized sections of the dish. We then mounted the heads of these workers on paper cards with double-sided tape and measured head width using a Leica M205 C stereo microscope (467 nm resolution) attached to a five megapixel Leica DFC 425 digital microscope camera. Head width (HW) is a widely used indicator of overall body size in ants [20, 56].

We estimated the average fat content of workers using a method established for *S*. *invicta* [38, 55]. We randomly selected ten workers from each replicate and dried specimens in an oven at 60°C for 48 hours. After 48 hours, we placed workers into 1.5mL microcentrifuge tubes (to prevent re-hydration) and then weighed each individual using a UMX2 microbalance with 0.1 μg resolution (Mettler-Toledo, Columbus, OH). Specimens were then placed in gelatin capsules and arranged in a Soxhlet Extractor for 24 hours with diethyl ether. After 24 hours, the ants were again dried in an oven at 60°C for several hours and were re-weighed on the microbalance. We estimated percent fat mass as ((dry mass − lean mass)/dry mass) [62].

### Statistical Analyses

Worker number and size were compared between treatments in a split-plot mixed model ANOVA with treatments (amino acids and carbohydrates) as fixed effects and colony as a random effect. Percent fat mass was compared between treatments in split-plot mixed model ANCOVA with fixed and random affects as above, and initial worker mass (pre-fat extraction) as a covariate. In this split-plot design a field colony represents a whole plot and one of the four experimental subcolonies is nested within the whole plot (representing a subplot). Additionally, because colony identity was significant in the model (Table 1), we only included replicates from field colonies that were represented in all four treatments at the end of the experiment (e.g., balanced design, n = 9). Worker number and percent fat mass were not normally distributed and were log_10_ and arcsine-root square transformed respectively. Analysis was completed in SAS software, version 9.4, SAS Institute, Cary, NC, USA.

**Table 1:** ANOVA table for the effect of diet treatment on worker number and worker body size.

**Worker Number**					
**Source**	**SS**	***df***	**MS**	***F***	***p***
Carbohydrates	0.1052	1	0.105	4.47	0.045
Amino Acids	0.0006	1	0.001	0.03	0.870
A. Acids & Carbohydrates	0.0118	1	0.012	0.50	0.490
Colony	3.1605	8	0.395	16.79	0.0001
Residual	0.5646	24	0.024	-	-
**Head Width**					
**Source**	**SS**	***df***	**MS**	***F***	***p***
Carbohydrates	0.006	1	0.006	5.82	0.024
Amino Acids	3.45 e^-5^	1	3.45 e^-5^	0.03	0.857
A. Acids & Carbohydrates	4.36 e^-5^	1	4.36 e^-5^	0.04	0.839
Colony	0.0961	8	0.012	11.65	0.0001
Residual	0.0248	24	0.001	-	-

In mature *S*. *invicta* colonies, workers can be divided into two distinct subpopulations, where workers with a HW > 0.75 mm are considered majors and workers with a HW < 0.75 mm are considered minors [27]. To improve resolution of where potential shifts in body size occur within the distributions we further subdivided the minors into two bins (0.5–0.675 mm and 0.675–0.75 mm) and the majors into two bins (0.75–1.125 mm and 1.125–1.5 mm) creating four equal size categories. To analyze variation in body size distributions among treatments, we used a G-test to compare the proportions of workers in each of the four size categories across treatments.

We tested for the existence of tradeoffs between the number of workers produced by a colony and mean worker body size (HW) using regression analysis for each treatment group (water, amino acids only, carbohydrates only, and amino acids and carbohydrates. To test for differences between slopes of treatments with worker number and treatment as fixed effects and colony identity as a random effect and corrected for multiple comparisons with a post-hoc Tukey test (PROC MIXED, SAS 9.4).

## Results

**Fig 1:**
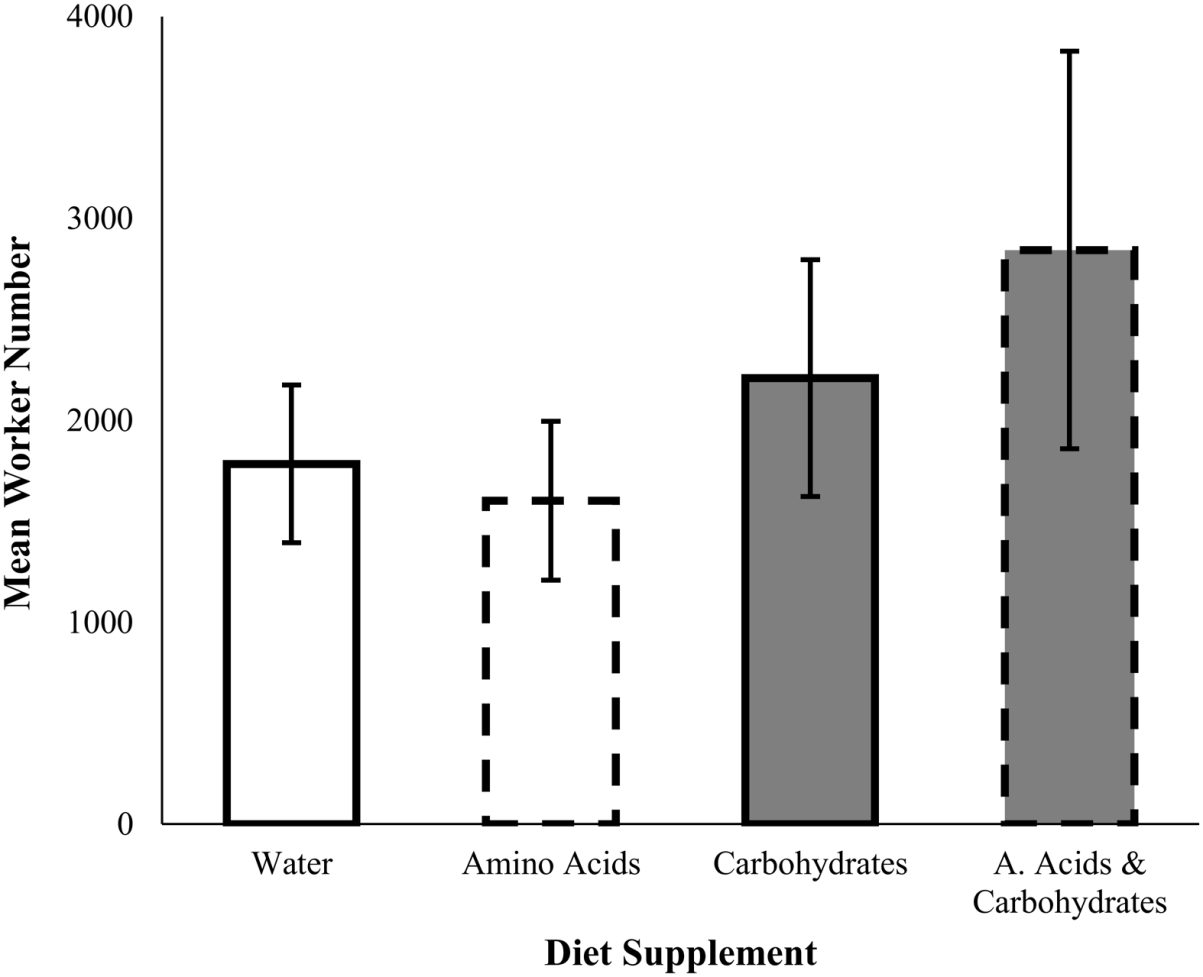
Mean (± SE) worker number for colonies supplemented with amino acids (dashed lines) and/or carbohydrates (grey bars) to their diet. Worker number was greater for colonies supplemented with carbohydrates compared to colonies not supplemented with carbohydrates (ANOVA F_*1*, *24*_ = 4.47, *p* = 0.045). There was no effect of amino acid supplementation on worker number (F_*1*, *24*_ = 0.03, *p* = 0.87).

Lab colonies supplemented with carbohydrates produced more workers compared to colonies not supplemented with carbohydrates (ANOVA, *F*_*1*, *24*_ = 4.47, *p* = 0.045) (Fig 1, Table 1). On average, colonies with access to carbohydrates produced ~50% more workers than colonies without access to carbohydrates. We found no effect of amino acid supplementation (*F*_*1*, *24*_ = 0.03, *p* = 0.87) or an amino acid and carbohydrate interaction (*F*_*1*, *24*_ = 0.50, *p* = 0.49) on worker number (Fig 1).

**Fig 2:**
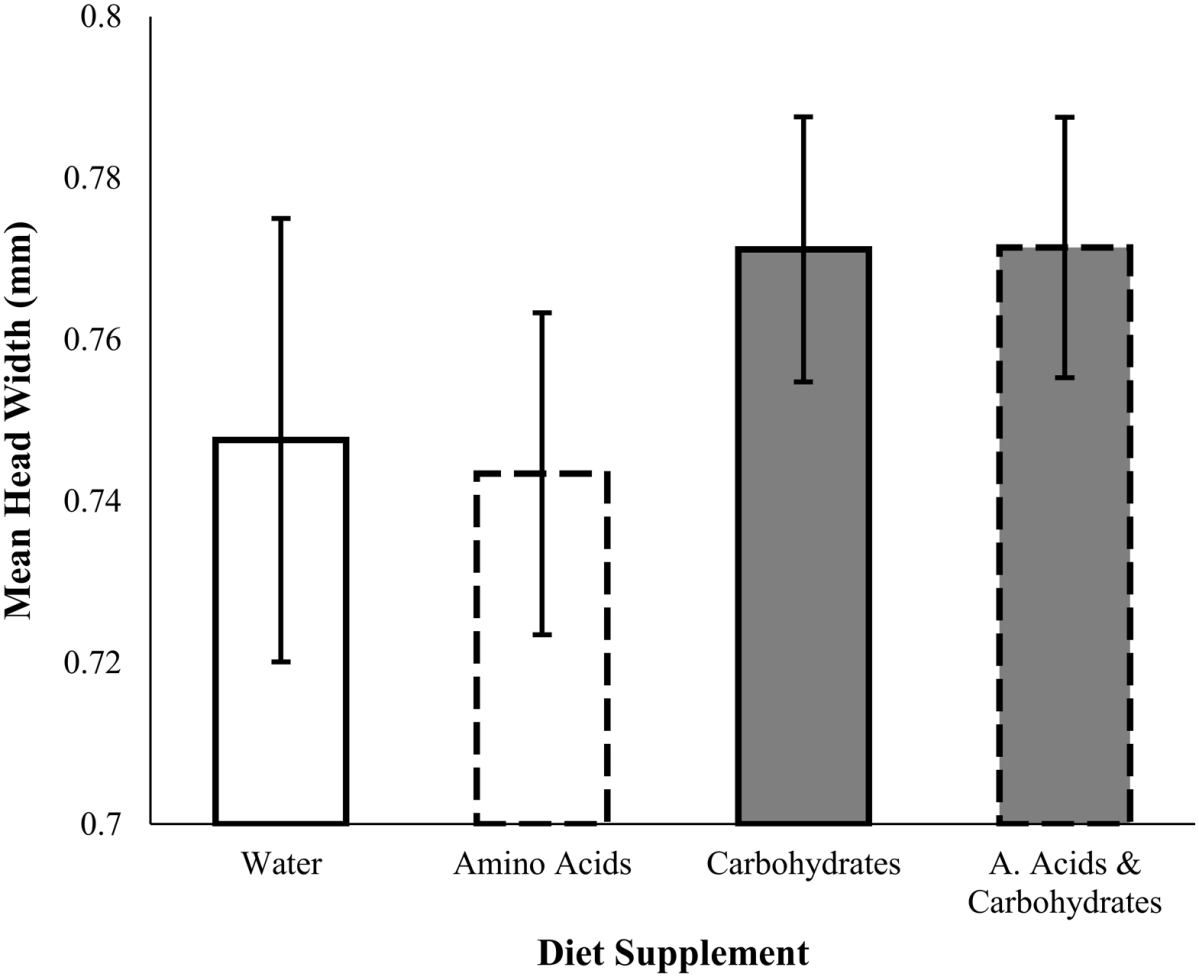
Mean (± SE) worker head width for colonies supplemented with amino acids (dashed lines) and/or carbohydrates (grey bars) to their diet. Head width was significantly larger in colonies supplemented carbohydrates than those not supplemented carbohydrates (ANOVA *F*_*1*, *24*_ = 5.82, *p* = 0.024). There was no effect of amino acid (*F*_*1*, *24*_ = 0.03, *p* = 0.86). There was no interaction between carbohydrates and amino acid supplementation on worker head width (*F*_*1*, *24*_ = 0.04, *p* = 0.84).

**Fig 3:**
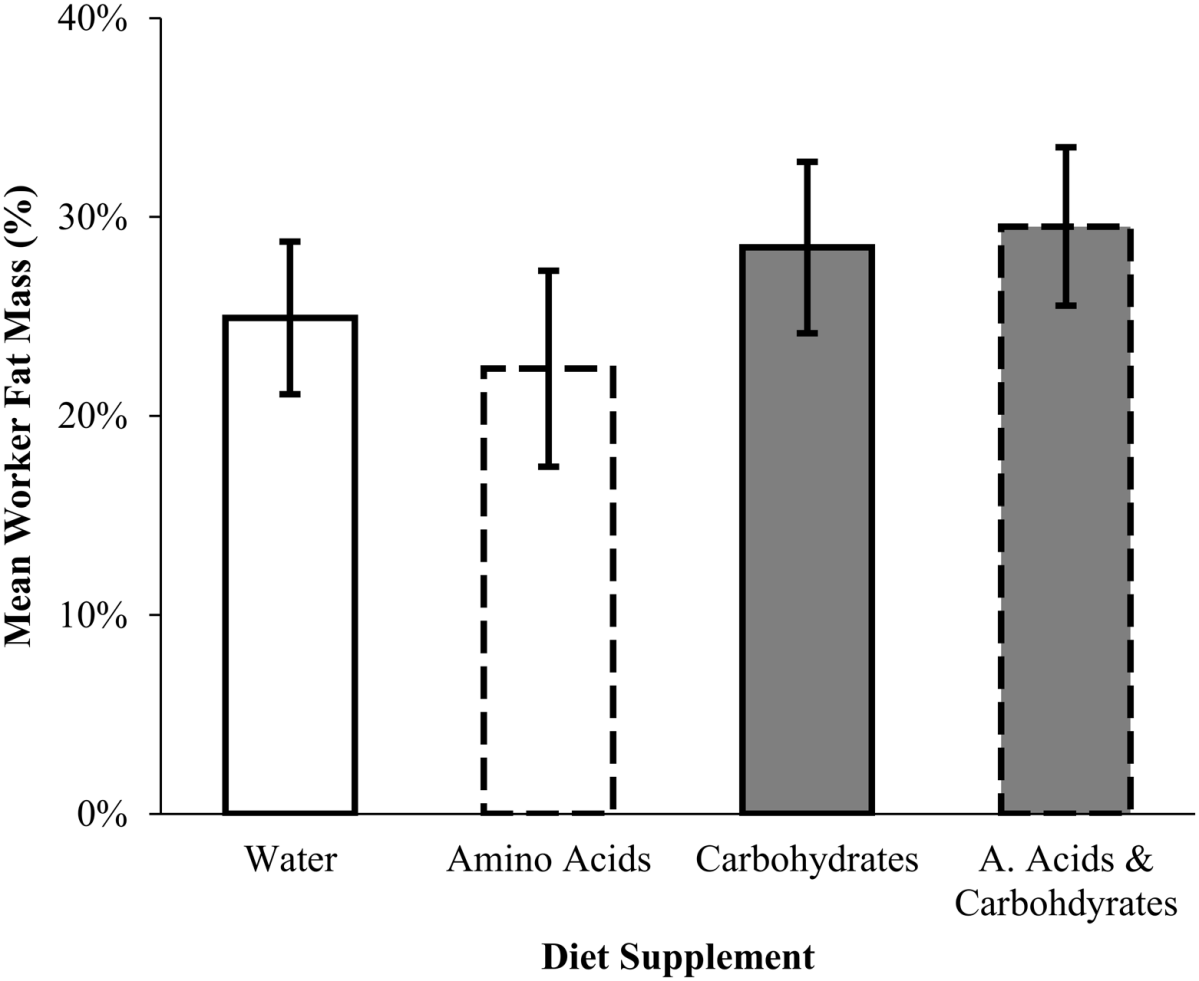
The mean (± SE) percent fat mass workers supplemented carbohydrates (gray bars) and/or amino acids (dashed lines) to their diet. There was no significant effect of carbohydrates (ANCOVA, *F*_*1*, *23*_ = 0.03, *p* = 0.86), amino acids (*F*_*1*, *21*_ = 0.01, *p* = 0.93), or their interaction (*F*_*1*, *24*_ = 0.1, *p* = 0.76) on worker fat mass.

Mean worker size (HW) was larger in colonies supplemented with carbohydrates compared to those of colonies not supplemented with carbohydrates (ANOVA, *F*_*1*, *24*_ = 5.82, *p* = 0.024) (Fig 2, Table 1). However, there was no effect of amino acid supplementation on worker size (*F*_*1*, *24*_ = 0.03, *p* = 0.86) (Fig 2), or an amino acid and carbohydrate interaction (*F*_*1*, *24*_ = 0.04, *p* = 0.84). The proportion of worker fat mass was independent of access to carbohydrates (ANCOVA, *F*_*1*, *23*_ = 0.03, *p* = 0.86), amino acids (*F*_*1*, *21*_ = 0.01, *p* = 0.93), and the interaction between the two macronutrients (*F*_*1*, *21*_ = 0.1, *p* = 0.76) (Fig 3).

**Fig 4:**
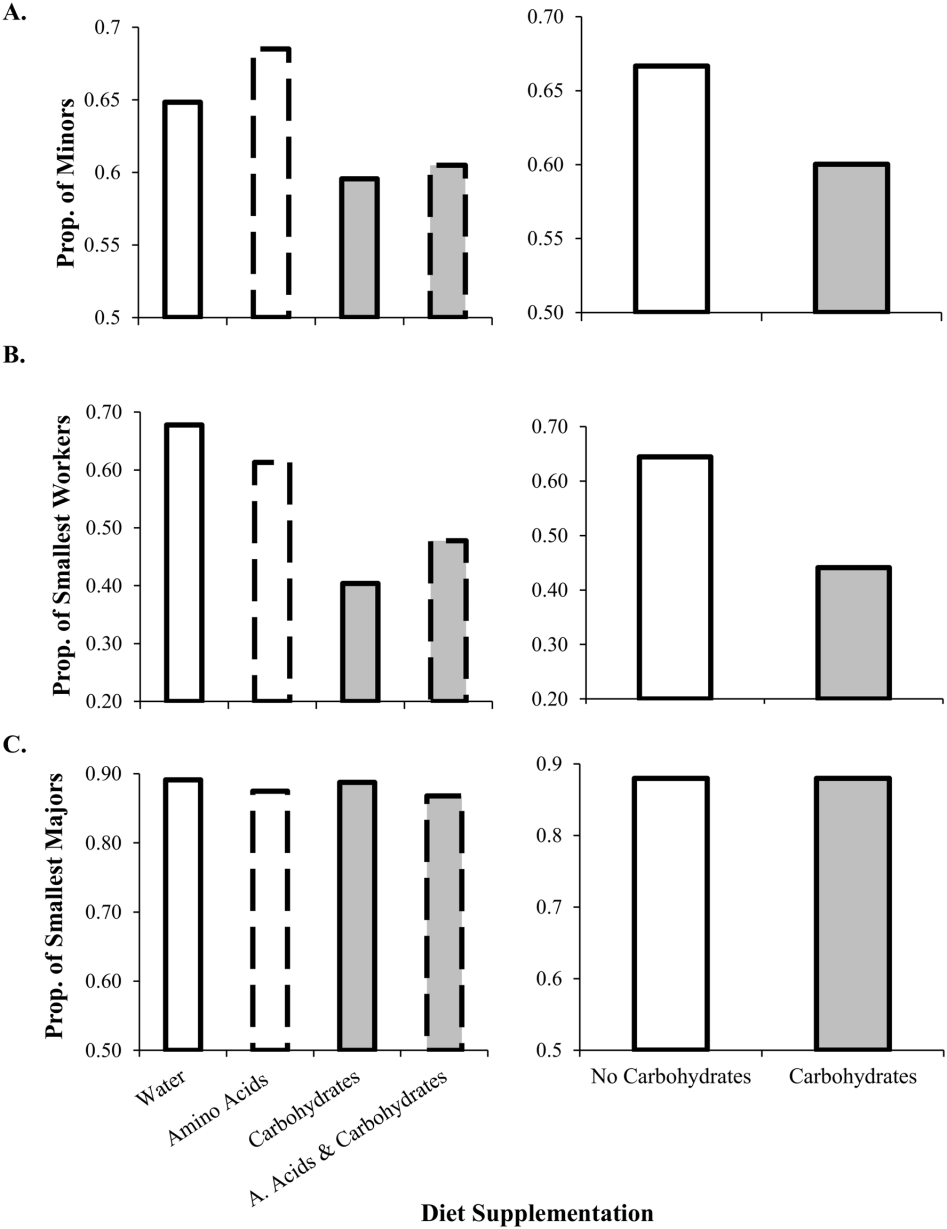
The proportion of workers from two subpopulations (minors = 0–0.75 mm and majors = 0.75–1.6 mm) (sensu Tschinkel 1988). **A.** There proportion of workers is significantly different between diet supplements (*G*-test *G =* 39.99, *d*.*f*. = 3, *p* < 0.0001). Specifically, there is a greater proportion of minor workers with wider HW from colonies reared with access to carbohydrates (gray bars) (*G =* 34.2 *d*.*f*. = 1, *p* < 0.0001). **B.** The proportion of smallest minor workers (0–0.675 mm) is significantly different between treatments (*G* = 314, *d*.*f*. = 3, *p* < 0.0001). Colonies with access to carbohydrates had significantly more workers with larger heads (*G* = 191, *d*.*f*. = 1, *p* < 0.0001). **C.** The proportion of the smallest major workers (0.75–1.125 mm) was not significantly different between treatments (*G* = 2.24, *d*.*f*. = 3, *p* = 0.52). There is no significant difference in HW of major workers from colonies reared without access to carbohydrates and those with access to carbohydrates (*G* = 0.2, *d*.*f*. = 1, *p* = 0.66).

**Fig 5:**
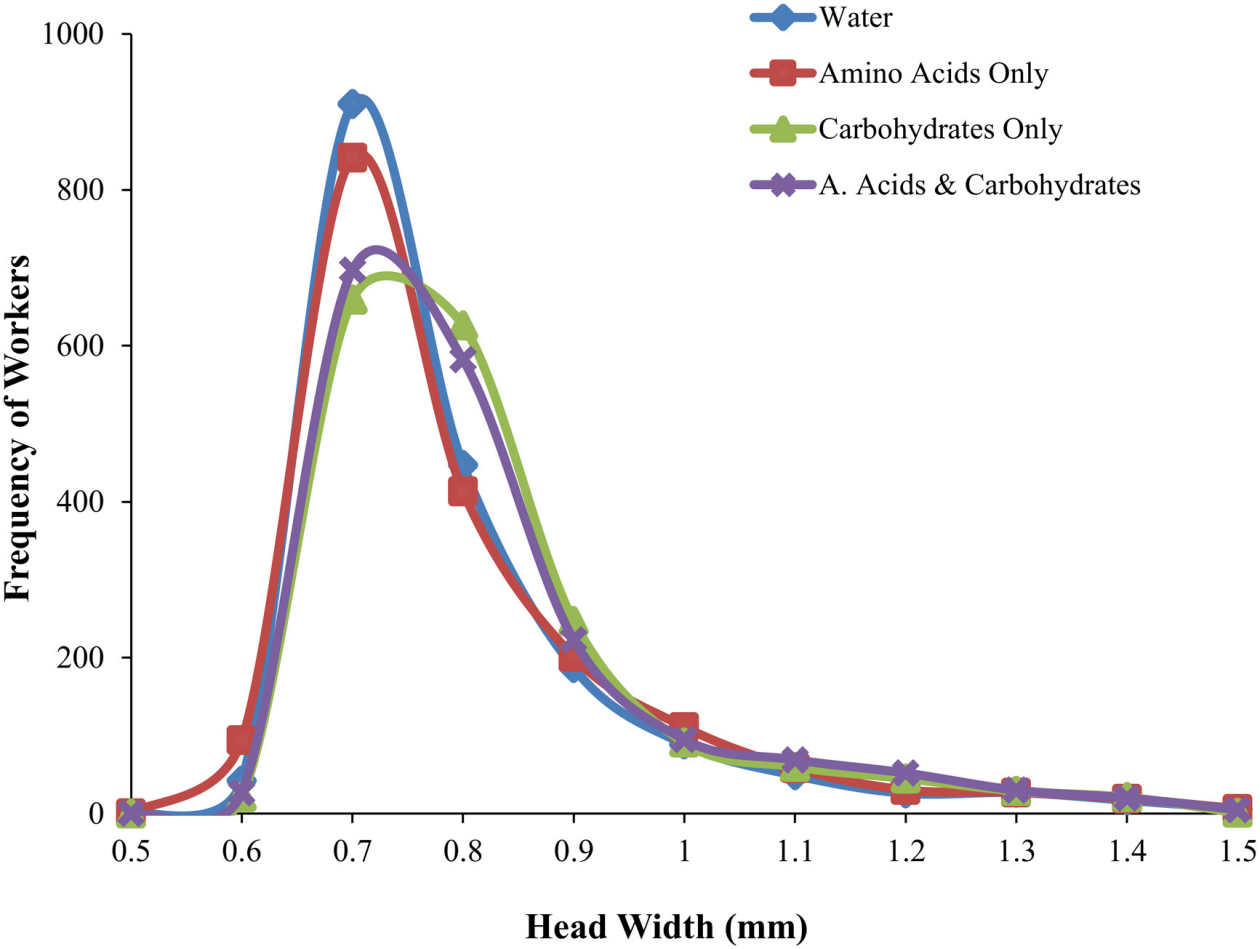
Worker body size distributions based on head width of experimental colonies of *Solenopsis invicta* after being reared for 60 days on diets that varied access to water (blue, diamonds) carbohydrates (green, triangles), amino acids (red, squares), or both (purple, asterisks).

The relative proportion of minor (0.5–0.75 mm) and major (0.75–1.5 mm) workers differed among diet treatments (*G =* 39.99, *df* = 3, *p* < 0.0001) (Fig 4A). When subdividing the worker caste into 4 categories, we also found differences between the proportions of “small minors” (0.5–0.675 mm) and “large minors” (0.675–0.75 mm) (*G* = 314.26, *df* = 3, *p* < 0.0001) (Fig 4B, Fig 5), but not between the proportions of “small majors” (0.75–1.125 mm) and “large majors” (1.125–1.5 mm) (*G* = 2.24, *df* = 3, *p* = 0.52) (Fig 4C, Fig 5). Colonies with access to carbohydrates produced more “large minors” (0.675–0.75 mm) compared to those without access to carbohydrates (*G* = 191.42, *df* = 1, *p* < 0.0001) (Fig 4B, Fig 5). There was no difference in the proportions of majors between colonies with access to carbohydrates versus colonies without access to carbohydrates (*G* = 0.20, *df* = 1, *p* = 0.66) (Fig 4C, Fig 5).

**Fig 6:**
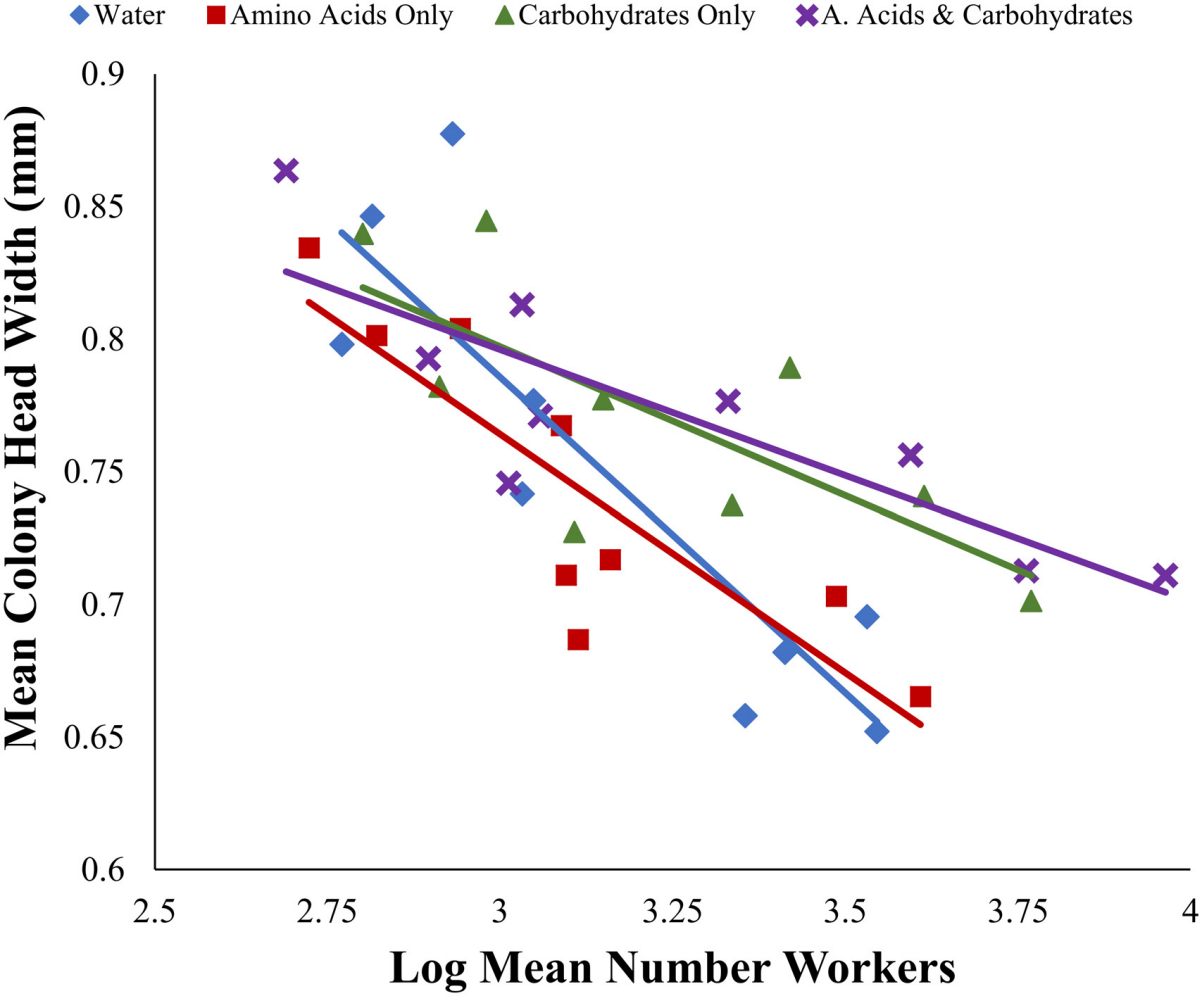
Relationship between worker number and mean worker size for colonies with access water (blue, diamonds), amino acids only (red, squares), carbohydrates only (green, triangles), and amino acids and carbohydrates (purple, X’s). The slope of colonies with access to amino acids and carbohydrate supplement was steeper than that of colonies with access to amino acid only (Tukey, *d*.*f*. = 22, *t* = -3.56, *p-adj* = 0.01) and water only (*d*.*f*. = 21, *t* = 2.90, *p-adj* = 0.04) supplements. The slope from colonies reared with a carbohydrate only supplement was steeper than colonies reared on amino acid only supplements (*d*.*f*. = 22, *t* = -3.41, *p-adj* = 0.01) but different from colonies reared with a water supplement (*d*.*f*. = 21, *t* = 2.73, *p-adj* = 0.057).

Across all treatments, mean colony worker number was negatively correlated with mean worker size (*p* < 0.05) (Fig 6, Table 2). The slope of colonies with access to amino acids and carbohydrate supplement was steeper than the slope for colonies with access to only amino acid (Tukey, *d*.*f*. = 22, *t* = -3.56, *p-adj* = 0.002) (Fig 6) and water supplements (*d*.*f*. = 21, *t* = 2.90, *p-adj* = 0.04). The slope from colonies reared with a carbohydrate only supplement was steeper than colonies reared on amino acid only supplements (*d*.*f*. = 22, *t* = -3.41, *p-adj* = 0.01), but not steeper than the slope from colonies reared with a water supplement (*d*.*f*. = 21, *t* = 2.73, *p-adj* = 0.057).

**Table 2:** Regression statistics for each treatment and the associated relationship between worker number and mean worker size.

Treatment	R-square	MSE	*d*.*f*.	*P*
Water	0.7732	0.04191	8	0.0018
Amino Acids	0.7518	0.02120	8	0.0029
Carbohydrates	0.5537	0.03519	8	0.0215
A. Acids & Carbohydrates	0.7098	0.02784	8	0.0044

## Discussion

Access to carbohydrate resources via ant-mutualists is thought to be a key factor in the ecological success of many ants by providing a reliable food source and the fuel required to maintain a larger number of high-tempo workers [35, 44, 45, 63]. Previous work revealed that lab colonies reared on high carbohydrate diets (or diets with a relatively high ratio of carbohydrates to amino acids) can have higher dry biomass of both workers and brood, and reduced worker mortality [39, 40, 43, 50, 64, 65]. Wilder and colleagues [40] reported that access to carbohydrates increased overall colony biomass (brood and workers) for the invasive, *S*. *invicta* in both the field and the lab. In this study, we found that differences in biomass in response to increased access to carbohydrates resulted from colony investment into both more and larger workers.

Increases in colony biomass supplemented with carbohydrates are largely thought to result from an increase in worker production and longevity [65–67]. We found that lab colonies of a polymorphic ant provided with access to carbohydrates also supported a greater number of “large minor” workers within the minor subcaste (0.5–0.75 mm) compared to those without access to carbohydrate resources. Observed differences in worker size therefore result from a shift in the mean body size of minor workers (i.e., the presence of more “medium” sized workers). Shifts in body size in response to diet supplementation have been previously observed in other ants including *Formica* [68] and *Pheidole* [69, 70]. In *S*. *invicta*, worker size is correlated with life span [16]. Therefore, the increase in worker size in the carbohydrate treatment likely contributes to an increase in colony biomass through two mechanisms; increase in the mass of individual workers and increase in worker longevity [43, 67], which is related to worker size [16]. We also found a negative relationship between worker number and worker size across all colonies, and the slope of this relationship was steeper for colonies without access to carbohydrate resources than those with access to carbohydrates. This pattern suggests that there are multiple strategies for increasing biomass, and that investment into either the number or size of individuals is subject to tradeoffs and influenced by macronutrient availability. Future research that varies the amount of resources, in addition to resource identity, will be useful for determining if this tradeoff is the result of resource limitation, or a result of colony demography.

We found a significant effect of colony identity on measures of size and worker number. This is likely the result of differences in colony age, condition, or genetics at the time of collection. For example, colony demography changes as a function of colony age [38, 42]. Therefore, age and size variation among source colonies likely influenced initial worker metrics, and these differences may have persisted through our 60-day experiment. Colony age in *S*. *invicta* is known to impact both worker size and the distribution of worker sizes with larger colonies typically producing larger workers [38, 40, 55]. The initial size of our experimental colonies was significantly smaller than that of mature *S*. *invicta* colonies, which can support over 300,000 workers [38, 42]. Initial worker number in this experiment may have artificially constrained the size of workers produced. Larger laboratory colonies than those used in the present study might have produced a shift in the size and number of workers within the major subpopulation (0.75–1.6 mm) [42]. Nonetheless, by standardizing the initial size of lab colonies and accounting for colony identity in our statistical models, we still found a significant response of worker size to diet.

Fat content increases with worker size in fire ants and larger workers may be used by the colony to store resources [38, 41, 42]. We did not find a significant difference in percent fat mass from colonies reared with or without access to carbohydrate resources. In contrast to our results, Grover and colleagues [46] found mean worker fat mass in Argentine ants (*Linepithema humile*) was two times greater in colonies provided with carbohydrate resources relative to colonies without carbohydrate resources. These differences may reflect general physiological differences in fat storage among ant species [71]. However, Grover et al. [46] also used a different method for estimating fat content [71]. Our methodology has been used previously with *S*. *invicta* [38, 42] and other ant species [72, 73], but it may not detect subtle differences among treatments. Furthermore, Tschinkel [38] found seasonal variation in worker percent fat mass in *S*. *invicta* with peak percent fat in mid-summer to fall. Our experiment was set up in the spring and it is possible that we would have more likely to see a change in percent fat mass among treatments if we ran our experiment longer. Worker percent fat mass may also remain static as colonies regulate their protein-to-carbohydrate ratio [43], and colonies with a carbohydrate supplement may have consumed a greater proportion of protein from the *ad libitum* crickets. Changes in worker fat content and storage is not only important to worker biomass, but is also linked to differences in behavior in ants [74, 75]. Therefore, future work would benefit from using multiple approaches for estimating fat content and worker condition in relation to diet or other factors.

In addition to fueling large colonies, access to carbohydrates may be particularly important for helping the establishment and success of small, incipient ant colonies [50, 76]. The importance of carbohydrate resources to worker size and growth suggests that small colonies with access to carbohydrate resources (e.g. extrafloral nectar or honeydew producing insects) pursue an allocation strategy that enhances production of workers in the “medium”- sized categories. Within introduced populations of *S*. *invicta*, colonies often feed more extensively on carbohydrate resources compared to colonies from native populations [59]. The importance of carbohydrate resources to patterns of worker production and survival is likely to result from differences in the digestive abilities of ant larvae and adult workers. Adult workers of *S*. *invicta*, for example, have a reduced digestive system compared to larvae and may carry solid foods to larvae for digestion [55, 77]. When only insect prey is available, larvae may divert resources away from their own growth to digest food for adult workers. This dynamic could influence larval growth and development, as well as limit the available energy to workers because larvae must balance nourishing themselves and feeding workers. Liquid carbohydrates, on the other hand, are relatively easy to digest by workers and satisfy their own energetic requirements without diverting resources away from developing larvae.

While access to carbohydrate resources increases colony biomass [40] and influences body size distributions (present study), all of our colonies were fed an *ad libitum* diet of crickets. The addition of crickets to colony diet likely reduces the impact of amino acid supplementation to worker number, size, and quality. If these colonies relied solely on the diet supplement treatments, it is likely that worker size, number, and overall worker quality would have also varied among individual treatments [39]. Improvements to artificial diets [66] provide opportunity to test how macronutrients influence colony investment into worker production. Future experiments that include more extreme variation in diet, and run for longer periods of time, will further elucidate the importance of macronutrients to colony investment into worker number and size [43, 64, 65].

Access to carbohydrate-rich resources appears to play an important role in determining allocation of resources in key life history traits (e.g., worker body size and body size distribution). Most studies on carbohydrate resources in ants have assumed they are used to fuel worker activity [78], aggression, or foraging [46, 79]. However, carbohydrate resources are also essential to the production of larvae [40, 43, 51, 64]. Larval development in ants and other insects is thought to be protein limited, but growing evidence suggests that carbohydrates are also needed for growth, particularly in holometabolous insects [64]. These results suggests that the traditional paradigms of ant nutrition, including colony investment into worker production is limited by protein may need to be reevaluated. Ants require specific concentrations of nutrients in particular ratios to maintain worker activity, colony growth, and worker mortality [43, 64]. Our results also show that nutritional ecology can have important implications for the success of introduced species like *S*. *invicta*. Alterations in the availability of plant-based resources through the monopolization of carbohydrate-rich resources (e.g., tending Hemipterans) may facilitate invasions into new environments because a larger and more numerous worker forces can overpower competitors [25, 54], better withstand temperature fluctuations [19], periods of food deprivation [80], and survive longer [16].
